# Phytohormone-ROS Crosstalk Regulates Metal Transporter Expression in *Sedum alfredii*

**DOI:** 10.3390/toxics13100823

**Published:** 2025-09-26

**Authors:** Shimiao Chen, Bin Shan, Yanyan Li, Fuhai Zheng, Xi Chen, Lilan Lv, Qinyu Lu

**Affiliations:** 1 Guangxi Subtropical Crops Research Institute, Nanning 530001, China; chenshimiao@gxaas.net (S.C.); zzbj219@163.com (B.S.); lvcancan1985@163.com (L.L.); 2 Key Laboratory of Quality and Safety Control for Subtropical Fruit and Vegetable, Ministry of Agriculture and Rural Affairs, Nanning 530001, China; 3 Guangxi Key Laboratory of Quality and Safety Control for Subtropical Fruits, Nanning 530001, China; 4 Fruit Tree Research Lab, Qinzhou Institute of Agricultural Sciences, Qinzhou 535000, China; liyanyan0202@163.com; 5 Agricultural Resources and Environmental Research Institute, Guangxi Academy of Agricultural Science, Nanning 530001, China; fuhaizheng110@126.com; 6 College of Food Science and Engineering, Henan University of Technology, Zhengzhou 450001, China; chenxi21221@gmail.com

**Keywords:** *Sedum alfredii*, phytohormones, cadmium accumulation, phytohormone-ROS crosstalk, heavy metal transporters

## Abstract

*Sedum alfredii* is a cadmium (Cd) hyperaccumulator, but the regulatory mechanisms linking phytohormones and redox balance to Cd transporter expression remain unclear. In this study, we omitted external cadmium (Cd) stress to isolate and examine the interplay between phytohormone and reactive oxygen species (ROS) signaling. Exogenous treatments with abscisic acid (ABA), indole-3-acetic acid (IAA), gibberellic acid (GA_3_), trans-zeatin (t-Z), and H_2_O_2_ were combined with analyses of hormone levels, antioxidant enzyme activities, and transporter gene expression. Correlation and PLS-SEM analyses identified the CAT–H_2_O_2_ module as a key node: ABA and IAA enhanced CAT activity and alleviated ROS-mediated repression of transporters, while GA_3_ and t-Z exerted opposite effects. Functional validation using an H_2_O_2_ scavenger revealed that the regulation of *HMA3* and *Nramp5* by ABA and t-Z is H_2_O_2_-dependent. In contrast, IAA modulates *Nramp5* through a ROS-independent pathway, while the regulatory effects of GA_3_ were negligible. Functional validation under Cd exposure suggests a model wherein *HMA3* and *Nramp5* act in a complementary manner to sequester and redistribute Cd in leaves, thereby supporting hyperaccumulation. These findings highlight hormone-specific ROS pathways as central to transporter regulation and provide mechanistic insights to improve phytoremediation efficiency.

## 1. Introduction

*Sedum alfredii* is a well-known hyperaccumulator of cadmium (Cd), lead (Pb), and zinc (Zn) that has been extensively used for phytoremediation of heavy metal-contaminated soils [1,2,3]. However, its overall remediation efficiency is constrained by the plant’s low biomass and slow growth rate, which are common characteristics of hyperaccumulator plants. Ref. [4], constraining the total amount of metal that can be extracted from soils. Therefore, enhancing heavy metal accumulation in the shoots of *S. alfredii* is a primary objective for improving its phytoremediation efficiency [5]. Therefore, achieving this goal requires a better understanding of the regulatory mechanisms governing heavy metal uptake, translocation, and accumulation in *S. alfredii*, including the identification of key signal transduction pathways and effective chemical agents to modulate these processes.

To date, multiple biological processes have been implicated in Cd hyperaccumulation by *S. alfredii*, including enhanced ion uptake across membranes [6,7], efficient xylem loading and translocation of metals [2,7], cellular sequestration and compartmentalization of Cd [8,9,10], and detoxification/chelation mechanisms [6,11]. These processes operate systematically and cooperatively, ultimately facilitating high Cd accumulation in the plant [12]. Phytohormones are increasingly recognized as essential regulators that coordinate many of these processes. For instance, the exogenous application of certain plant growth regulators, such as indole-3-acetic acid (IAA), brassinolide (BR), and abscisic acid (ABA), has been shown to enhance Cd phytoextraction by boosting the antioxidant defense system and reducing lipid peroxidation damage [13]. This evidence suggests that hormonal signaling, often intertwined with redox regulation, plays a crucial role in the accumulation of heavy metals.

Various phytohormones (such as auxins, brassinosteroids, ABA, salicylic acid, jasmonic acid, and ethylene) can modulate heavy metal uptake and tolerance through multiple mechanisms. These hormones regulate the transcription of metal transporter genes [14,15]; modulate root system architecture, thereby influencing metal uptake [16]; enhance antioxidant capacity to mitigate metal-induced oxidative stress [14,15]; and engage in crosstalk with one another to fine-tune plant stress responses [14,16]. Concurrently, heavy metal stress is closely associated with reactive oxygen species (ROS) signaling in plants. Cd, as a non-essential toxic element, induces oxidative stress that can damage cells and inhibit growth [17,18]. Previous studies have shown that S. alfredii displays minimal ROS accumulation at ≤0.1 mM CdCl_2_, whereas exposure to ≥0.2 mM CdCl_2_ triggers marked oxidative stress responses, including increased H_2_O_2_ and O_2_^−^ production as well as antioxidant enzyme activation [8]. This threshold-like shift indicates that rising Cd levels rapidly induce ROS signaling, which may interfere with hormone-regulated pathways. However, ROS also function as important secondary messengers in stress signaling and can influence metal uptake processes. For example, elevated ROS levels can affect the expression of metal transporter genes such as *IRT1* and *NRAMP*, which mediate the uptake and transport of divalent metal cations, including Cd [7]. Thus, oxidative signaling is thought to contribute to the regulation of Cd uptake and translocation, potentially playing a role in *S. alfredii*’s hyperaccumulation capacity. Moreover, many phytohormones can trigger ROS production and activate downstream defense pathways [19,20]. The extensive crosstalk between hormonal signals and ROS enables plants to orchestrate transcriptomic and metabolic adjustments under stress [19,20]. This hormone–ROS network is mediated by diverse signaling components, including ROS-generating enzymes like NADPH oxidases [19], mitogen-activated protein kinase cascades [21], stress-responsive transcription factors [19], and secondary signaling molecules such as flavonols [22].

Despite these insights, the precise regulatory interplay between heavy metal stress, ROS, and phytohormones remains poorly understood. One major challenge is disentangling the direct effects of heavy metals from the signaling interactions among hormones and ROS. In our previous study [12], we found that exogenous ABA can have a dual effect on Cd accumulation in *S. alfredii* depending on the stress intensity. Under moderate Cd exposure—associated with relatively low ROS levels—ABA enhanced Cd uptake and root-to-shoot translocation by upregulating the expression of divalent cation transporter genes. In contrast, under high Cd stress (with elevated ROS), this promotive effect was diminished or even reversed [12]. These results imply that a basal level of ROS is required for ABA to stimulate Cd accumulation, pointing to a critical ABA–ROS crosstalk in regulating metal transporter activity. However, as these experiments were conducted in the presence of Cd, it was difficult to distinguish responses driven by Cd-induced stress from those activated by hormone-specific signaling.

In the present study, we addressed this knowledge gap by removing external Cd treatment to eliminate direct heavy metal stress signals. This experimental design enabled us to isolate the interactions between phytohormones and ROS in regulating Cd-related transporters, thereby eliminating the confounding influence of concurrent Cd toxicity. We specifically examined how ABA, in the absence of external Cd, influences the expression of Cd transporter genes in *S. alfredii* via ROS-mediated signaling. By working under Cd-free conditions, we aimed to clarify the role of ABA–ROS crosstalk in the signaling network underlying Cd uptake and accumulation. To our knowledge, this is the first attempt to investigate the regulatory interactions between hormones and ROS in a hyperaccumulator plant without applying heavy metal stress, providing a novel perspective on the mechanisms of Cd hyperaccumulation. The insights gained from this work shed light on how phytohormone-ROS signaling regulates heavy metal transport and may ultimately guide new strategies for enhancing phytoremediation efficiency through targeted manipulation of plant signaling pathways.

## 2. Materials and Methods

### 2.1. Materials Preparation

*S. alfredii* (HE) seeds, collected from the ancient lead-zinc mining area in Quzhou, Zhejiang Province, were bred in the nursery. Cuttings with uniform growth, approximately 2 cm in height, were selected. Cuttings were hydroponically cultured for one month to induce rooting, following the method described by Lu et al. [12]. Then, phytohormones and H_2_O_2_ were applied to the leaves as a foliar spray at the doses specified in Table 1. After 6 h, the shoots of each treatment were harvested, flash-frozen in liquid nitrogen, and stored at −80 °C for subsequent processing.

### 2.2. Gene Expression Analysis

Expressions of related genes were analyzed using quantitative PCR (qPCR) according to Lu et al. [12]. Each 0.1 g sample from the treatments was homogenized with liquid nitrogen, and RNA was extracted using the Plant Total RNA Isolation Kit (Sangon Biotech, Co., Ltd., Shanghai, China), followed by reverse transcription to cDNA using the MightyScript First Strand cDNA Synthesis Master Mix (Sangon Biotech). Gene expression was then measured using specific primers (see Table S1) and the TaqProbe 2X qPCR-Multiplex (Sangon Biotech) according to the manufacturer’s instructions on a CFX96 Touch Real-Time PCR Detection System (Bio-Rad Laboratories, Hercules, CA, USA). The relative expression of target genes under various treatments was calculated to determine differential expression.

### 2.3. Endogenous Hormone Content Analysis

The concentrations of indoleacetic acid (IAA), trans-zeatin (t-Z), gibberellin (GA_3_), and abscisic acid (ABA) were measured using enzyme-linked immunosorbent assay (ELISA) [25]. A total of 5 g of each treatment sample was ground with liquid nitrogen and dissolved in 80% methanol, shaking at 4 °C overnight for extraction. Next, the mixtures were subjected to refrigerated centrifugation at 10,000× *g* for 10 min. The supernatants were then concentrated to near dryness using nitrogen to remove methanol and subsequently redissolved in the phosphate-buffer solution for further analysis. Indirect ELISAs were performed using kits provided by Yuanju Bio (Hangzhou, China). The optical densities were then acquired using a microplate reader (Tecan Infinite E Plex, Männedorf, Switzerland).

### 2.4. Reactive Oxygen Species Analysis

The concentrations of hydrogen peroxide (H_2_O_2_) and superoxide anion (O_2_^−^) were measured using the methods described by Sunil and Narayana [26] and Choi et al. [27], respectively. Each 0.1 g sample was homogenized using liquid nitrogen and then dissolved in 80% methanol, with shaking at 4 °C overnight for extraction. The mixtures were then subjected to refrigerated centrifugation at 10,000× *g* for 10 min. For H_2_O_2_ determination, the supernatants were treated with 1 mL of potassium iodide and 1 mL of 2 M hydrochloric acid, resulting in the liberation of iodine, which bleaches toluidine blue, with absorbance measured at 628 nm. For O_2_^−^ measurement, phagocytic cells were incubated with 0.1% nitroblue tetrazolium (NBT), lysed, and the blue formazan deposits dissolved using 2 M potassium hydroxide and dimethylsulfoxide. The absorbance of the dissolved formazan was then measured at 620 nm. The optical densities for both assays were acquired using the spectrophotometer (T9CS, PERSEE, Beijing, China).

### 2.5. Antioxidant Enzyme Activity Analysis

Antioxidant enzyme activities, including peroxidase (POD), superoxide dismutase (SOD), and catalase (CAT), were measured by the increase in guaiacol oxidation, inhibition of nitroblue tetrazolium (NBT) photochemical reduction, and decomposition of hydrogen peroxide, respectively. Each 0.5 g sample was homogenized in 5 mL of pre-cooled phosphate buffer (pH 7.0) and centrifuged at 12,000× *g* for 20 min to obtain the crude enzyme in the supernatant. The activities were determined using POD, SOD, and CAT Activity Assay Kits (Sangon Biotech), and the optical densities were measured with a spectrophotometer (T9CS, PERSEE) at 470, 560, and 240 nm, respectively. These wavelengths were selected according to standard protocols, as they correspond to the characteristic absorption peaks of the respective reaction products (guaiacol oxidation at 470 nm for POD, NBT photoreduction at 560 nm for SOD, and H_2_O_2_ decomposition at 240 nm for CAT) [28]. All enzyme activities were expressed as units per mg of protein, with protein concentrations determined using the Bradford method.

### 2.6. Cd Treatment Experiment and Measurements

To functionally validate the hormone–ROS–transporter pathways inferred from the PLS-SEM model under Cd-free conditions, we conducted a subsequent cadmium treatment experiment with an H_2_O_2_ scavenger.

Cd validation experiments were conducted using the same hydroponic system described in Section 2.1, with three biological replicates per treatment. Plantlets were exposed to a nutrient solution containing 0.1, 0.2, 0.3, 0.4, or 0.5 mM CdCl_2_ for 6 h. Each Cd level was split into two groups: one supplemented with 1 mM dimethylthiourea (DMTU, an H_2_O_2_ inhibitor) and one without. Shoots were harvested separately, washed with 5 mM Na_2_EDTA and deionized water to remove surface-bound Cd, oven-dried at 70 °C, and ground for metal analysis.

Cd concentration was determined using atomic absorption spectrophotometry (AAS, Thermo Scientific ICE 3500, Waltham, MA, USA) after digestion of ~0.1 g dried tissue with concentrated HNO_3_ in a closed-vessel microwave digestion system (EXPEC 790, EXPEC Technology, Hangzhou, China).

Endogenous H_2_O_2_ levels were measured spectrophotometrically using the KI method (as described in Section 2.4 on ROS assays). Phytohormone contents (ABA, IAA, GA_3_, t-Z) were quantified by ELISA following Section 2.3, and *HMA3*/*Nramp5* transcript levels were determined by qPCR as in Section 2.2.

### 2.7. Data Analysis

Analysis of variance (ANOVA) followed by Duncan’s multiple-range post hoc test was conducted using SPSS 22 Statistics (IBM, Armonk, NY, USA). PLS-SEM was performed using SmartPLS 4 (SmartPLS GmbH, Bönningstedt, Germany). Graphs were created using Origin 2022 software (OriginLab, Northampton, MA, USA). Correlation analysis and heat maps were generated in R (version 4.3.2; R Core Team) using the corrplot package. To validate hormone–ROS–transporter interactions, a moderation analysis was implemented in R. Linear models were fitted, and robust standard errors were obtained with the sandwich and lmtest packages. Interaction effects were visualized and simple slope analyses performed using the interactions package. For each hormone–transporter pair, Fisher’s r-to-z comparisons were conducted with the cocor package to test whether correlations differed significantly between inhibitor and non-inhibitor conditions.

## 3. Results

### 3.1. The Impact of Exogenous Treatments on Cd Transporter Gene Expression

The expression of genes related to Cd accumulation is shown in Table 2. Compared with CK, all transporters exhibited significant responses to exogenous phytohormones and H_2_O_2_, with the strongest regulation observed for *ZIP2* and members of the *Nramp* and *HMA* families.

Within the *Nramp* family, low-dose t-Z caused a strong induction of *Nramp3* (2.29 vs. 1.00 in CK) and *Nramp6* (2.81 vs. 1.00), while high-dose t-Z suppressed both genes (*Nramp3* 0.47; *Nramp6* 0.41). A notable dose-dependent divergence was observed under ABA treatment: A high dose of ABA strongly favored *Nramp3* (2.22) and *Nramp6* (3.44), while a low dose had a milder effect. Similarly, IAA low-dose induced *Nramp3* (2.09) and *Nramp6* (1.91).

The *HMA* family was highly sensitive to exogenous stimuli but showed distinct patterns among members. High-dose treatments of t-Z, ABA, GA_3_, and H_2_O_2_ consistently enhanced *HMA2* and *HMA4*. For example, ABA low-dose enhanced *HMA2* (2.75) and *HMA4* (2.45), and GA_3_ low-dose strongly promoted *HMA2* (3.88) and *HMA4* (4.26). The most substantial increases were observed under high-dose H_2_O_2_ treatment, with *HMA2* and *HMA4* expression levels reaching 5.41-fold and 6.15-fold, respectively, relative to the control (CK). In contrast, while all treatments suppressed *HMA3* expression, the effect was least pronounced under low-dose IAA and low-dose t-Z, with relative expression levels of 0.90 and 0.72, respectively.

Among all genes tested, the *ZIP2* transporter exhibited the most dramatic expression changes, with its transcript levels increasing by orders of magnitude under specific treatments (Table 2). From a baseline expression of nearly 1.0 in CK, it increased to 39.9 under t-Z low-dose, 158.7 under ABA high-dose, 87.6 under GA_3_ low-dose, and 71.1 under GA_3_ high-dose. Even under milder treatments such as IAA low-dose, *ZIP2* was strongly upregulated (29.6), highlighting it as the most hormone-responsive transporter in this dataset. By contrast, *ZIP3* was generally suppressed, with values falling below CK under most treatments (e.g., 0.26 under ABA high-dose). For *IRT1*, expression was usually reduced relative to CK; however, IAA at high doses significantly upregulated *IRT1* (1.73 vs. 1.00), suggesting a dose-specific redirection of Cd uptake routes under auxin influence.

In summary, the response to hormone treatment was highly gene-specific. Low-dose treatments often preferentially induced *Nramp* transporters (*Nramp3*/*6*), whereas high-dose treatments, particularly high-dose H_2_O_2_, strongly promoted *HMA2* and *HMA4*. Across all genes, *ZIP2* was the most significantly upregulated, showing increases of two to three orders of magnitude.

### 3.2. Effects of Exogenous Hormone Application on Endogenous Phytohormones

**Figure 1:**
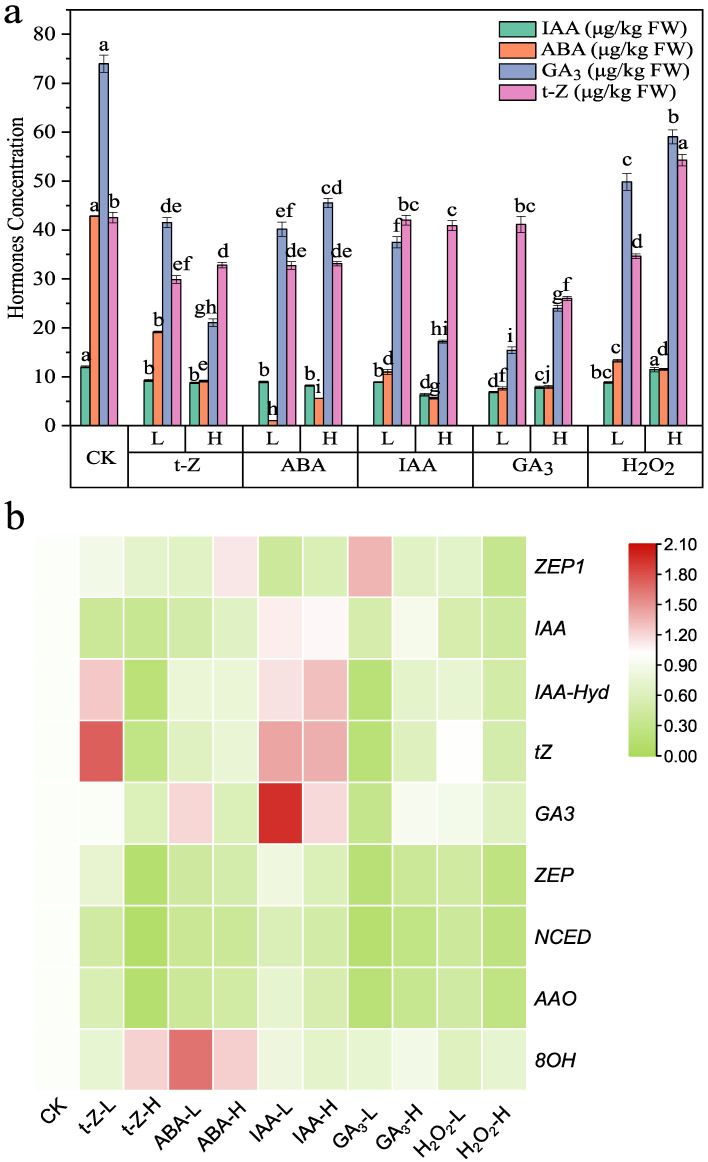
The concentrations of endogenous hormones and the expression of metabolizing enzymes in the shoot of *S. alfredii*. (**a**). The hormone concentrations in the shoot, Orange, pink purple, cyan green, and blue purple bars represent the concentration of IAA, ABA, GA3, and t-Z. (**b**). The heatmap of gene expression across different treatments, red denotes higher expression, while green denotes lower expression. Error bars represent the standard error of the mean (SE). Different lowercase letters above the bars indicate significant differences among varieties (ANOVA, *p* < 0.05).

The effects of endogenous phytohormone contents in Shoots are shown in Figure 1a, Table S2. Raw values for Figure 1a. Most treatments resulted in a general decline in endogenous phytohormone levels, although the extent varied among hormones. The most potent suppression was observed for endogenous ABA, the accumulation of which was nearly eliminated by high-dose applications of either ABA or GA_3_ (5.6 μg/kg and 7.9 μg/kg, respectively, vs. 42.9 μg/kg in CK). GA_3_ levels were likewise highly sensitive, dropping from 74 μg/kg in CK to 21.1 μg/kg under high-dose t-Z and 17.2 μg/kg under high-dose IAA, while H_2_O_2_ treatments also reduced GA_3_ to 49.8 μg/kg (low-dose) and 59.0 μg/kg (High-dose). IAA showed a more moderate decline, decreasing from 12.0 μg/kg in CK to ~9.3 μg/kg under low-dose t-Z and 7.9 under high-dose GA_3_, with the most potent inhibition under high-dose IAA (6.3 μg/kg). In contrast, t-Z was less consistently suppressed; although most treatments lowered its content to ~30–33 μg/kg, high-dose H_2_O_2_ significantly promoted it to 54.3 μg/kg, ~28% above CK. Collectively, these results indicate that exogenous hormone applications generally downregulated endogenous phytohormone pools. Among them, ABA and GA_3_ levels were the most significantly reduced. The magnitude of inhibition was often dose dependent, while specific treatments, notably high-dose H_2_O_2_, deviated from this trend by enhancing t-Z despite reducing ABA and GA_3_. In general, inhibitory effects were dose-dependent, with higher concentrations producing more substantial reductions. However, exceptions existed, such as high-dose H_2_O_2_, which significantly elevated t-Z despite decreasing ABA and GA_3_.

### 3.3. Effects of Exogenous Treatments on Phytohormone-Related Metabolic Enzymes

The expression patterns of phytohormone-related metabolic enzymes in shoots are shown in Figure 1b. In the ABA biosynthetic branch (*ZEP/NCED/AAO*), most treatments suppressed transcript levels relative to CK. Consistent with this overall trend, *ZEP1* was reduced under several conditions (e.g., t-Z low-dose ≈ 0.87; t-Z high-dose ≈ 0.71; low-dose ABA ≈ 0.66; H_2_O_2_ high-dose ≈ 0.33 vs. CK ≈ 1.00), whereas GA_3_ low-dose produced an opposite effect with a significant increase of *ZEP1* to ~1.35. This dose-dependent divergence highlights that GA_3_ at low levels can relieve the repression of ABA biosynthesis, while oxidative or cytokinin inputs tend to down-modulate it.

In the ABA catabolic branch, ABA 8′-hydroxylase (*ABA8′OH*) was generally repressed across treatments, although notable exceptions occurred under ABA (both low- and high-dose) and high-dose t-Z, which induced its expression (e.g., ABA low-dose ≈ 1.65; ABA high-dose ≈ 1.24; t-Z high-dose ≈ 1.23). This suggests that ABA turnover is generally reduced under most conditions, but can be enhanced by specific hormonal cues.

Within the IAA metabolic module, most exogenous treatments downregulated the IAA biosynthetic marker (e.g., t-Z low-dose ≈ 0.40; t-Z high-dose ≈ 0.35; ABA low-dose ≈ 0.49; GA_3_ low-dose ≈ 0.51; H_2_O_2_ high-dose ≈ 0.42), whereas IAA low-dose (≈1.10) and IAA high-dose (≈1.06) promoted its expression, consistent with an auxin-mediated positive feedback on its own synthesis. In the IAA catabolic arm, *IAA-Hyd* was selectively upregulated by t-Z low-dose and both IAA doses, but remained repressed or unchanged under most other treatments; notably, ABA, H_2_O_2_, and t-Z high-dose preferentially suppressed IAA synthesis more than *IAA-Hyd*, while both GA_3_ doses showed the opposite tendency (i.e., relatively more potent effects on the catabolic side).

For t-Z metabolism, t-Z synthetase was induced under IAA treatments (both doses), but was suppressed by t-Z high-dose and most other treatments. Increasing the dose of t-Z or H_2_O_2_ generally intensified the inhibition of t-Z synthetase, whereas GA_3_ displayed a reverse dose–response trend, with stronger promotion emerging at higher concentrations.

Finally, GA_3_ synthetase showed hormone-specific and dose-dependent regulation: IAA treatments and ABA low-dose enhanced expression, while t-Z (especially high-dose), ABA high-dose, H_2_O_2_, and GA_3_ high-dose led to significant suppression. Overall, low-dose applications tended to exert mild promotion or partial maintenance of GA_3_ biosynthesis, whereas high-dose inputs more frequently drove repression.

Together, these data indicate that exogenous cues reshape hormone biosynthesis and catabolism in a dose- and hormone-specific manner. ABA biosynthesis is broadly repressed (with GA_3_ low-dose as a key exception), while *ABA8′OH*-mediated catabolism is context-dependent—generally reduced but inducible by ABA and t-Z. Auxin imposes positive feedback on its own synthesis.

### 3.4. Effects of Exogenous Treatments on Reactive Oxygen Species (ROS) and Related Metabolic Enzymes

**Figure 2:**
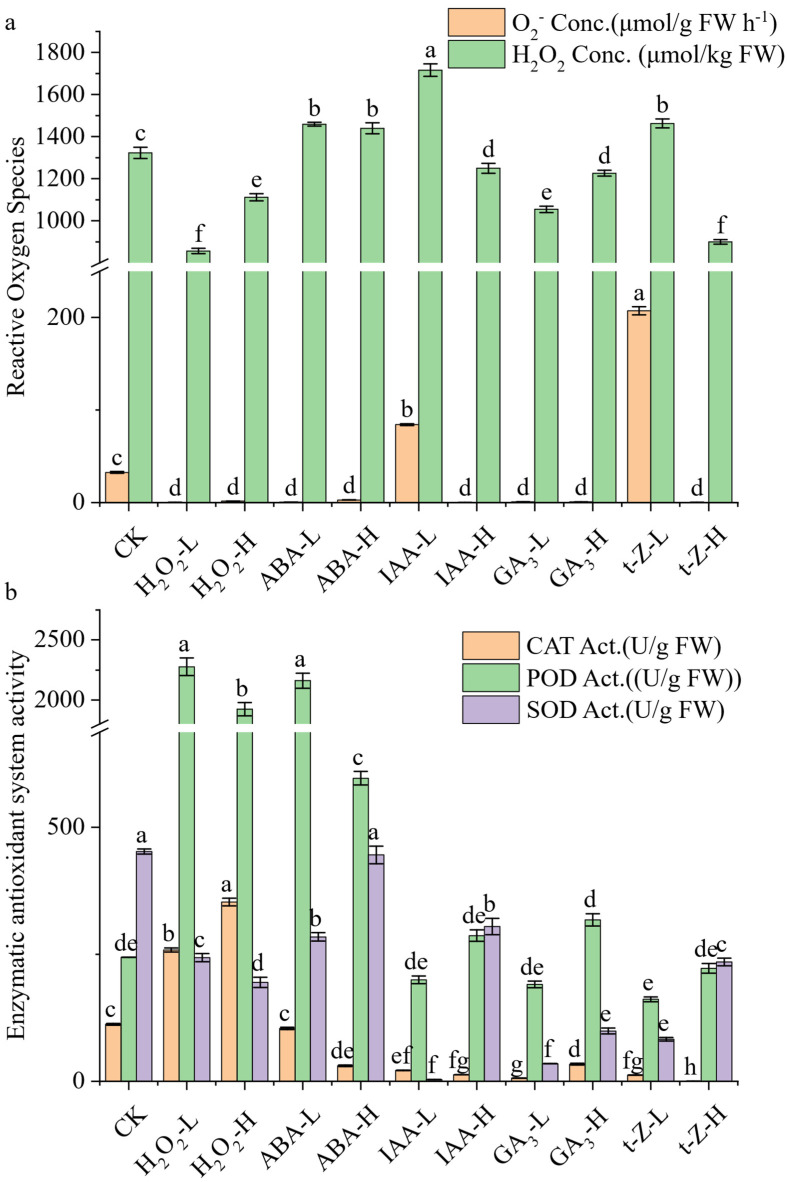
The Responses of Endogenous Reactive Oxygen Species (**a**) and Associated Antioxidant Enzymes (**b**) to Exogenous Treatments. (**a**) Light green, and light orange bars represent the concentration of O_2_^−^ and H_2_O_2_. (**b**) Light orange, light green, and light purple bars represent the activities of CAT, POD and SOD, respectively. Error bars represent the standard error of the mean (SE). Different lowercase letters above the bars indicate significant differences among varieties (ANOVA, *p* < 0.05).

The responses of endogenous ROS and associated antioxidant enzymes to exogenous treatments are shown in Figure 2.

The basal production rate of O_2_^−^ in the control (CK) group was 32.6 μmol g^−1^ FW h^−1^. Several treatments significantly lowered this rate; notably, low-dose H_2_O_2_ and high-dose t-Z decreased the production to nearly undetectable levels (0.24 μmol g^−1^ FW h^−1^ and 0.27 μmol g^−1^ FW h^−1^, respectively). In stark contrast, other treatments markedly elevated the O_2_^−^ production rate. Low-dose IAA markedly elevated O_2_^−^ to 84.3 μmol g^−1^ FW h^−1^, while low-dose t-Z induced the strongest promotion, reaching 207.2 μmol g^−1^ FW h^−1^ over six times the CK value.

Regarding H_2_O_2_ accumulation, CK shoots contained 1323 μmol kg^−1^ FW. Low-dose IAA significantly increased H_2_O_2_ to 1716 μmol kg^−1^ FW, while low-dose ABA (1459 μmol kg^−1^ FW) also caused a slight but significant increase. Conversely, high-dose t-Z reduced H_2_O_2_ to 900 μmol kg^−1^ FW, and both GA_3_ treatments lowered it to 1055 μmol kg^−1^ FW (low-dose) and 1226 μmol kg^−1^ FW (high-dose). Exogenous H_2_O_2_ treatments also decreased endogenous H_2_O_2_, with low-dose H_2_O_2_ at 857 μmol kg^−1^ FW and high-dose H_2_O_2_ at 1112 μmol kg^−1^ FW.

Antioxidant enzyme activities showed substantial divergence among treatments. SOD activity in CK was 452 U g^−1^ FW, but most treatments suppressed it drastically, with low-dose IAA showing the most severe inhibition (3.6 U g^−1^ FW). High-dose IAA partially restored SOD (305 U g^−1^ FW), whereas high-dose ABA (446 U g^−1^ FW) remained nearly unchanged relative to the control. POD activity in CK was 244 U g^−1^ FW, but treatments with low-dose H_2_O_2_ (2276 U g^−1^ FW) and low-dose ABA (2161 U g^−1^ FW) caused nearly tenfold increases. High-dose H_2_O_2_ (1922 U g^−1^ FW) and high-dose ABA (597 U g^−1^ FW) also enhanced POD relative to CK, though to a lesser extent. IAA and GA_3_ treatments remained below 320 U g^−1^ FW, while t-Z showed intermediate effects (161–222 U g^−1^ FW). CAT activity was 112 U g^−1^ FW in CK. Low-dose and high-dose H_2_O_2_ treatments elevated CAT to 258 U g^−1^ FW and 353 U g^−1^ FW, respectively, more than doubling the control. Conversely, CAT activity was strongly inhibited by most hormonal treatments. Specifically, activity levels were reduced to 31 U g^−1^ FW under high-dose ABA, 21 U g^−1^ FW with low-dose IAA, 6 U g^−1^ FW with low-dose GA_3_, and a mere 5.3 U g^−1^ FW under high-dose t-Z, indicating profound enzymatic suppression.

Collectively, these results reveal a dual regulatory pattern: specific treatments (e.g., low-dose IAA, t-Z) promoted ROS accumulation, whereas others (e.g., H_2_O_2_, GA_3_, high-dose t-Z) enhanced ROS scavenging capacity by stimulating POD and CAT activities.

### 3.5. Correlation Analysis Between Endogenous Phytohormones, Antioxidant Enzymes, and Metal Transporter Genes

**Figure 3:**
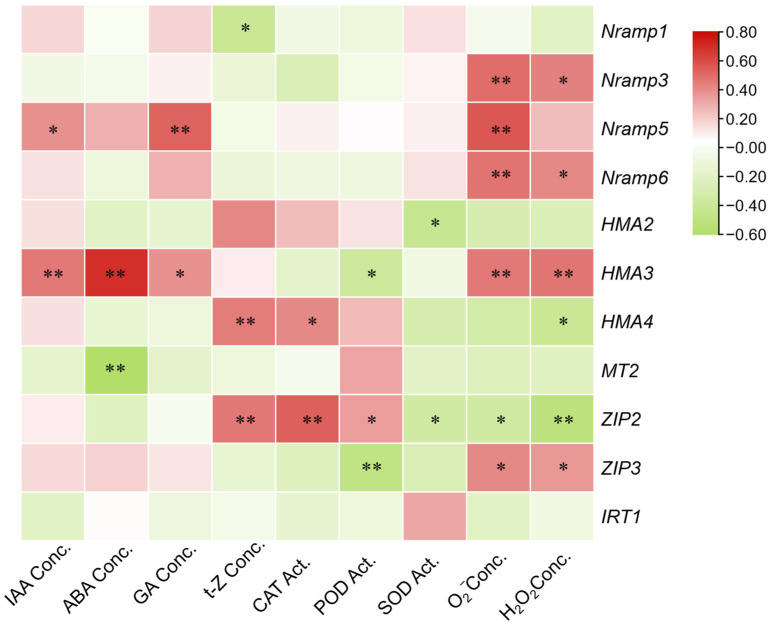
Correlation Analysis of Endogenous Phytohormones, Antioxidant Enzymes, and Metal Transporter Genes. Red denotes positive correlations, and green denotes negative correlations, with color intensity indicating the magnitude of the correlation coefficient. Significance levels: * *p* < 0.05; ** *p* < 0.01.

To further elucidate the regulatory network among endogenous phytohormones, antioxidant enzymes, and metal transporter genes, correlation analyses were conducted (Figure 3). In the first correlation matrix, phytohormones exhibited strong internal coherence. IAA and GA displayed a highly significant positive correlation (r = 0.895, *p* < 0.01), while ABA was also positively correlated with IAA (r = 0.696, *p* < 0.01) and GA (r = 0.622, *p* < 0.01). Moreover, t-Z showed significant positive correlations with IAA, ABA, and GA (r = 0.355–0.419, *p* < 0.05). By contrast, antagonistic patterns were observed between hormones and antioxidant enzymes. ABA was negatively correlated with SOD (r = −0.442, *p* < 0.01), and GA was negatively correlated with POD (r = −0.569, *p* < 0.01). In addition, antioxidant enzymes displayed strong synergy, as evidenced by positive correlations between POD and SOD (r = 0.405, *p* < 0.05) and between POD and CAT (r = 0.777, *p* < 0.01). These findings suggest that phytohormones tend to accumulate in a coordinated manner, whereas their increase often coincides with a reduction in antioxidant enzyme activities.

In the second correlation matrix, diverse associations were observed between phytohormones and genes encoding metal transporters. IAA was negatively correlated with *Nramp1* (r = −0.439, *p* < 0.05), *Nramp5* (r = −0.399, *p* < 0.05), and *HMA3* (r = −0.407, *p* < 0.05), but positively correlated with *MT2* (r = 0.374, *p* < 0.05). ABA exhibited the strongest influence, showing a highly significant negative correlation with *HMA3* (r = −0.648, *p* < 0.01), while positively correlating with *MT2* (r = 0.774, *p* < 0.01) and *ZIP2* (r = 0.567, *p* < 0.01). Similarly, GA was negatively correlated with *Nramp1* (r = −0.432, *p* < 0.05), *Nramp5* (r = −0.596, *p* < 0.01), and *Nramp6* (r = −0.448, *p* < 0.05). By contrast, t-Z exhibited only weak or non-significant correlations with most transporters. Collectively, these results highlight that ABA plays a central role in shaping transporter expression, particularly by suppressing *HMA3* while enhancing *ZIP2*, whereas IAA and GA predominantly act to inhibit *Nramp*-mediated metal uptake.

### 3.6. Integrated Multivariate Analysis (PLS-SEM) of Transporters, Hormones, and ROS

**Figure 4:**
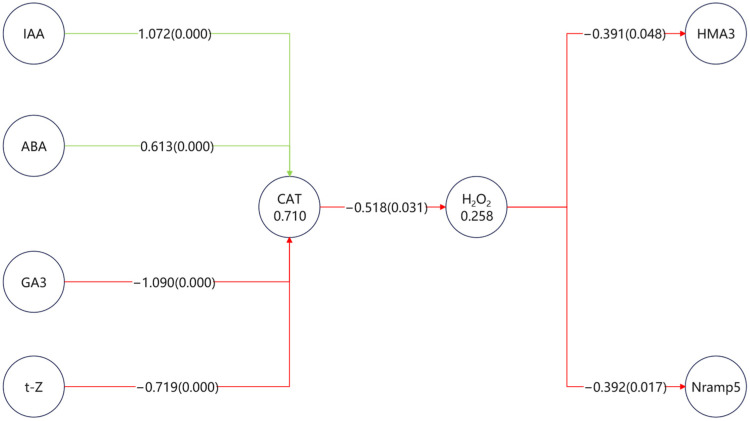
Partial least squares structural equation model (PLS-SEM) highlighting significant pathways linking hormones, ROS metabolism, and transporter expression. Only significant paths connecting hormones to metal transporters are shown. Green arrows represent positive effects, while red arrows indicate negative effects. Numbers on the paths denote standardized path coefficients, with values in parentheses indicating *p*-values.

All measured parameters, including hormone metabolic enzymes, hormone levels, antioxidant enzyme activities, ROS, and transporter expression, were incorporated into a PLS-SEM framework to reconstruct potential regulatory pathways. The complete model is provided in the Supplementary Materials (Figure S1), while Figure 4. highlights only the significant paths that directly connect hormones (via ROS metabolism) to transporters.

The results revealed a CAT–H_2_O_2_ axis as the central regulatory hub, through which multiple hormones indirectly shaped transporter expression. Hormones exerted contrasting effects on CAT activity: IAA (+1.07) and ABA (+0.61) acted as positive regulators, while GA_3_ (−1.09) and t-Z (−0.72) served as negative regulators. These opposing inputs determined the ROS balance, with elevated CAT activity suppressing H_2_O_2_ accumulation (−0.52).

Downstream, ROS exerted a consistent negative effect on transporter expression, significantly reducing *Nramp5* (−0.39, *p* = 0.017) and *HMA3* (−0.39, *p* = 0.048). Thus, positive hormonal inputs (IAA, ABA) alleviated ROS pressure and indirectly favored transporter regulation, whereas negative inputs (GA_3_, t-Z) promoted ROS accumulation and ultimately repressed transporter activity.

In terms of relative magnitude, GA_3_ showed the strongest suppression, while IAA had the strongest stimulation, indicating that auxin and gibberellin represent the dominant but antagonistic forces within this network.

### 3.7. Moderation Analysis of Hormone–Transporter Relationships by H_2_O_2_ Inhibition

To validate the SEM-inferred hormone–ROS–transporter pathways, we performed a moderation analysis using H_2_O_2_ inhibition (Table 3). The manipulation check confirmed that the inhibitor effectively reduced H_2_O_2_ accumulation in a Cd-dependent manner. Although the overall inhibitor main effect was not significant (β = −0.031, *p* = 0.128), a potent inhibitor × Cd Level interaction (β = −0.371, *p* < 0.001) indicated that H_2_O_2_ accumulation was significantly suppressed under high Cd conditions. These patterns are consistent with the SEM structure, in which CAT lowers H_2_O_2_ and H_2_O_2_ negatively regulates *HMA3* and *Nramp5*.

Simple slope analyses revealed distinct patterns of hormone–transporter regulation. For *HMA3*, ABA (*p* = 0.048) and t-Z (*p* = 0.02) showed significant negative effects in the absence of inhibitor, which became non-significant under inhibition, suggesting that both hormones act through an H_2_O_2_-dependent pathway. In contrast, GA_3_ and IAA had no significant effect on *HMA3* in either condition. For *Nramp5*, ABA exhibited a striking reversal: a significant negative effect without inhibitor (*p* = 0.01) shifted to a significant positive effect under inhibition (*p* < 0.01). t-Z again showed a significant negative association without the inhibitor (*p* < 0.001), which disappeared with inhibition. IAA displayed a robust positive effect on *Nramp5* across both groups (*p* < 0.001), with nearly identical slopes, indicating an H_2_O_2_-independent mechanism. GA_3_ had no significant influence on *Nramp5*.

Fisher’s r-to-z tests further confirmed these interaction patterns. Significant group differences in correlations were detected for ABA–*HMA3*, ABA–*Nramp5*, t-Z–*HMA3*, and t-Z–*Nramp5*, while IAA–*Nramp5* remained consistently positive across groups, and GA_3_–transporter pairs showed no significant differences. Finally, leaf Cd concentrations varied among treatments (Supplementary Table S3). Across Cd doses (0.1–0.5 mM), application of the H_2_O_2_ inhibitor generally resulted in higher Cd accumulation compared to the corresponding non-inhibitor controls (e.g., from 15.2 to 33.9 at 0.1 mM Cd). Although Cd accumulation reflects multiple pathways beyond those captured by the SEM, the consistent increase under inhibition supports the functional relevance of H_2_O_2_-mediated regulation of Cd handling in leaves. Detailed measurements of endogenous hormone contents, H_2_O_2_ concentrations, and transporter expression levels are provided in Supplementary Tables S4–S6 for reference.

## 4. Discussion

The regulation of metal transporter expression involves intricate interactions among heavy metals, ROS, and phytohormones, and the precise signaling pathways remain largely unresolved [29,30]. Previous studies have demonstrated that Cd levels strongly affect how phytohormones influence Cd uptake and translocation [12,13], making it difficult to decouple direct hormonal or ROS effects from Cd-induced responses. In this study, Cd itself was excluded from the treatments to minimize this interference and to investigate the extent to which phytohormones and ROS alone influence the expression of Cd-related transporters in *S. alfredii*. Our findings provide correlative evidence suggesting that hormone–ROS interactions could be important modulators of transporter expression, thereby offering candidate pathways for further functional investigation.

PLS-SEM analysis identified phytohormones as central regulators of Cd uptake and accumulation, acting either positively or negatively depending on the treatment. Among them, H_2_O_2_ and CAT emerged as pivotal mediators that bridge hormonal signals with Cd transporter responses, particularly *HMA3* and *Nramp5*. ABA, IAA, GA_3_, and t-Z displayed contrasting roles in shaping Cd transporter expression. SEM highlighted that ABA and IAA exerted a consistent positive impact on leaf catalase (CAT) activity, whereas t-Z and GA_3_ suppressed CAT. This hormone-specific regulation underscores CAT as an early functional node in the hormonal control of Cd handling in leaves. Previous studies support such trends, showing that ABA can enhance CAT activity under stress conditions [31,32], and that auxin–antioxidant crosstalk contributes to stress adaptation [31]. In contrast, the observed suppression of CAT by t-Z and GA_3_ is consistent with their roles in balancing growth and stress responses [33]. Together, these observations indicate that ABA and IAA function as positive regulators of CAT in leaves, whereas t-Z and GA_3_ act as suppressors, reflecting a hormone-driven modulation of antioxidant readiness.

Within this framework, leaf CAT acts as a proximal regulatory switch linking hormone inputs to Cd-transporter transcription. Treatments that increased CAT (ABA, IAA) were associated with maintained or elevated expression of *HMA3* and *Nramp5*, while reduced CAT (t-Z, GA_3_) coincided with transporter repression. This observation aligns with our SEM model, in which CAT-mediated detoxification of H_2_O_2_ alleviates its inhibitory pressure on both *HMA3* and *Nramp5*, thereby allowing for sustained transporter expression. Thus, hormone-driven CAT elevation relieves ROS-mediated repression of transporter genes, while CAT suppression reinforces it. This interpretation aligns with established roles of catalase in maintaining intracellular H_2_O_2_ homeostasis and the broader recognition of H_2_O_2_ as a transcription-modulating signal in plant stress responses, including heavy metal exposure [34,35].

At the transporter level, the SEM highlighted that H_2_O_2_ negatively regulates both *HMA3* and *Nramp5*, but their functions in leaves are complementary rather than antagonistic. Upregulation of *HMA3* reflects enhanced vacuolar sequestration of Cd in mesophyll or storage cells—a hallmark strategy of hyperaccumulators that enables high shoot Cd accumulation while buffering cytosolic toxicity [36]. In contrast, while *Nramp5* is classically considered a root uptake transporter, emerging evidence suggests it can contribute to metal partitioning in aerial tissues; for example, *OsNramp5* in rice leaf sheaths mediates Mn unloading from the xylem [37]. By analogy, elevated *Nramp5* expression in *S. alfredii* leaves may facilitate Cd distribution into safe compartments, synergizing with *HMA3*-mediated sequestration. Together, these two transporters define a leaf-centric Cd accumulation module, in which *HMA3* enhances vacuolar storage and *Nramp5* supports controlled cellular distribution.

Taken together, these interlinked mechanisms establish a coherent framework: hormones modulate CAT, CAT modulates transporter expression, and transporters cooperate to compartmentalize Cd in leaves. This layered model explains how *S. alfredii* achieves effective shoot-level Cd accumulation through a hormone–CAT–transporter cascade, highlighting the leaf as a central site for coordinated regulation of Cd buildup.

To test the robustness of the SEM-derived relationships, we employed an intervention-based moderation design that perturbed the ROS node. These tests partially validated the relationships inferred from the SEM, though some discrepancies were observed. Specifically, hormone effects on *HMA3*/*Nramp5* that were significant in the SEM emerged as H_2_O_2_-dependent only for ABA and t-Z (their effects vanished under H_2_O_2_ inhibition), whereas IAA’s positive association with *Nramp5* persisted under inhibition, and GA_3_ showed no robust impact. By contrast, the negative influence of H_2_O_2_ on both transporters remained consistent with the SEM. Taken together, the intervention results narrow the SEM: they support a core pathway in which ABA and t-Z stimulate H_2_O_2_ accumulation that subsequently suppresses *HMA3* and *Nramp5*, while revealing that IAA–*Nramp5* regulation can occur independently of H_2_O_2_. This pattern is mechanistically plausible because H_2_O_2_ is a central stress signal that integrates with multiple hormone pathways [38], and ABA can upregulate catalase isoforms to buffer H_2_O_2_, whereas cytokinins can promote ROS production in specific tissues and contexts [39].

Methodologically, these discrepancies are expected. PLS-SEM is a variance-based, prediction-oriented composite approach; it estimates conditional associations under a prespecified model but does not, by itself, establish causal effects or guarantee that an association will survive perturbation of an internal node (here, H_2_O_2_). Endogeneity, omitted variables, and unmodeled interactions (e.g., the strong Cd dose × inhibitor effect we observed) can yield significant paths that do not generalize under intervention [40]. Moderation analyses—by directly testing slope changes across perturbed vs. unperturbed conditions—probe when a relation holds and therefore often constrain SEM-based hypotheses [41]. Our findings thus support the use of SEM as a hypothesis-generating tool, with intervention-based tests serving to subsequently refine the model by identifying and removing non-robust pathways (retain ABA/t-Z ROS-dependent links), flag IAA–*Nramp5* as ROS-independent, and deem GA_3_ effects negligible under the tested regime.

Biologically, the hormone-specific divergence we uncovered is also consistent with the literature: (i) H_2_O_2_ sits at the hub of metal stress signaling (MAPKs, redox-sensitive TFs), so blocking it should attenuate hormone effects that route through ROS [38]; (ii) ABA can elevate catalase expression/activity and thereby prevent excessive H_2_O_2_ accumulation, aligning with its H_2_O_2_-dependent footprint here [39]; (iii) cytokinin (t-Z) can induce ROS (e.g., in guard cells) and thus reasonably shows H_2_O_2_-dependent transport regulation [38]; and (iv) some auxin outputs can be routed via parallel (ROS-independent) modules, explaining the IAA–*Nramp5* persistence under inhibition.

While PLS–SEM provides valuable conditional insights by isolating direct effects under controlled model architecture, it remains inherently limited in capturing the full richness of hormone–hormone interactions, especially when feedback loops, dose sensitivity, and metabolic conversions are involved.

Our expression profiles reveal that IAA is not significantly associated with the expression of hormone-metabolizing enzymes (e.g., IAA synthases or hydrolases) under non-IAA treatments. However, IAA treatments themselves selectively induced IAA-hydrolase, suggesting a self-regulatory turnover mechanism to prevent overaccumulation. This aligns with the notion that auxin homeostasis is maintained by tight metabolic feedback loops rather than broad cross-hormonal control.

By contrast, t-Z (a cytokinin) consistently suppressed ABA biosynthetic enzymes, reflecting potential antagonism, which is consistent with patterns observed by O’Brien et al. [42], where cytokinin–ABA crosstalk modulates development and stress adaptation in a context-dependent manner. Meanwhile, ABA appears to suppress GA_3_ biosynthesis, and vice versa, showcasing their classical antagonism in balancing growth and stress responses. This relationship aligns with findings reviewed in Emamverdian et al., where the interplay between heavy metal stress-associated ABA and GA directs protective versus growth-promoting outcomes [31]. Furthermore, GA_3_ displayed dose-dependent metabolic control: low-dose GA_3_ partly enhanced its biosynthesis, whereas high-dose treatment led to suppression—highlighting a concentration-sensitive regulation fitting recent reviews on GA_3_ crosstalk dynamics under stress [43].

Together, these patterns support a model of hormone crosstalk operating via metabolic modulation, including mutual antagonism and self-regulation, rather than through broad direct activation or suppression. This observation underscores that complex crosstalk is more clearly revealed at the level of enzyme expression, even in the absence of significant pairwise correlations. It also suggests that hormones self-regulate within a layered network, which aligns with their distinct roles in stress adaptation.

A striking finding of this study was the dramatic upregulation of *ZIP2*, which far exceeded that of any gene within the core CAT–H_2_O_2_ pathway. Its exclusion from the PLS-SEM framework is therefore highly informative, strongly suggesting the existence of a parallel, ROS-independent regulatory network that is highly sensitive to hormonal cues. The discovery of this distinct pathway thus opens a novel avenue for future research. While correlation analysis revealed notable associations between several phytohormones (e.g., IAA, ABA, GA_3_, t-Z) and transporter genes, such as *ZIP* family members (e.g., *ZIP2*, *ZIP6*) and *NRAMPs* (e.g., *Nramp1*), these hormone–transporter pairs did not emerge as significant in the PLS-SEM framework. This discrepancy suggests that their regulation is likely mediated by alternative molecular pathways rather than the CAT–H_2_O_2_ signaling module. For example, *ZIP* transporters—responsible for the uptake and redistribution of essential and non-essential metals, such as Zn, Fe, Mn, and Cd—are often regulated by metal-responsive transcription factors and hormonal cues, independent of ROS signaling. Structural control of these genes is typically mediated by *bZIP*-related transcriptional networks responsive to metal homeostasis rather than oxidative stress signals [44]. Similarly, *NRAMP* family proteins, although classically associated with root uptake, include members such as tobacco *NtNRAMP3*, which is preferentially expressed in the leaf xylem and likely functions in local metal unloading to maintain homeostasis. This expression pattern underscores its potential regulation by developmental or metal-status signals rather than ROS fluctuations [45]. Moreover, evolving evidence supports the involvement of hormone-responsive transcription factors (e.g., *NAC*, *WRKY*, *MYB* families) in directly influencing transporter gene expression. These TFs often respond to hormonal stimuli or metal stress through regulatory cascades distinct from ROS-mediated pathways [46]. Lastly, hormonal effects on transporters may be context- or dose-specific, only evident under particular physiological conditions. Such nuanced regulatory trends may yield detectable correlations but may not be strong or consistent enough to emerge as direct pathways in the PLS-SEM model, which prioritizes robust and dominant interactions.

This study offers new insights into the hormone-ROS signaling network in *S. alfredii*; however, the precise molecular mechanisms require further investigation. Future research can be extended on both the mechanistic and application fronts. At the mechanistic level, the priority will be to identify the key molecular components of the H_2_O_2_ signaling pathway, such as its upstream sensor proteins and downstream protein kinase cascades, to construct a complete signal transduction chain. At the application level, our findings offer potential targets for enhancing phytoremediation efficiency. We propose that genetic engineering or chemical priming approaches could be used to strategically bolster the antioxidant system and signaling responsivity of *S. alfredii*. Such efforts could optimize its bioaccumulation capacity and environmental resilience, thus advancing its potential in the remediation of contaminated soils.

## 5. Conclusions

This study establishes that phytohormones and ROS interact as an integrated signaling network to fine-tune cadmium transporter expression in *S. alfredii*. By removing external Cd stress, we demonstrated that hormone-induced responses are primarily mediated through redox homeostasis. Central to this regulation is the CAT–H_2_O_2_ module, identified via PLS-SEM as a key hub through which hormones exert opposing effects: ABA and IAA enhance CAT activity to alleviate H_2_O_2_-mediated repression of *HMA3* and *Nramp5*, whereas GA_3_ and t-Z suppress CAT, amplifying oxidative inhibition. Functional validation using H_2_O_2_ scavenging, however, revealed hormone-specific pathway dependencies: ABA and t-Z regulate transporters in an H_2_O_2_-dependent manner, IAA modulates *Nramp5* independently of ROS, and GA_3_ shows minimal regulatory influence. These findings underscore a model wherein *HMA3* and *Nramp5* act synergistically to compartmentalize Cd in leaves, thereby facilitating hyperaccumulation. Our work provides a mechanistic framework for hormone–ROS–transporter crosstalk, highlighting the potential for manipulating these pathways to enhance phytoremediation efficiency.

## Figures and Tables

**Table 1:** Application Dosages of Exogenous Hormones and H_2_O_2_ in *S. alfredii* Treatments.

	CK	ABA (mg/L)	IAA (mg/L)	GA_3_ (mg/L)	t-Z (mg/L)	H_2_O_2_ (mg/L)
L	deionized water	100	50	100	50	25
H		200	100	200	100	50

Note: The concentrations of exogenous phytohormones and H_2_O_2_ were designed based on differences in endogenous levels reported in our previous studies on *S alfredii* under Cd and ABA treatments [23,24]. The low concentration treatment (L) corresponded to the reported difference value, while the high concentration treatment (H) was set as twice the difference value.

**Table 2:** The expression of the heavy metal transporter in the shoot of *S. alfredii*. Values represent three biological replicates’ mean ± standard error (SE). Different lowercase letters within a row indicate significant differences among varieties (ANOVA, *p* < 0.05). The same lettering convention is used in subsequent tables.

Gene Expression	CK	t-Z		ABA		IAA		GA_3_		H_2_O_2_	
		L	H	L	H	L	H	L	H	L	H
*Nramp1*	1.00 ± 0.08 ^b^	0.43 ± 0.14 ^c^	0.11 ± 0.01 ^c^	0.56 ± 0.19 ^c^	0.40 ± 0.04 ^c^	0.22 ± 0.06 ^c^	0.18 ± 0.03 ^c^	0.11 ± 0.01 ^c^	1.54 ± 0.31 ^a^	0.34 ± 0.02 ^c^	0.16 ± 0.01 ^c^
*Nramp3*	1.00 ± 0.11 ^c^	2.29 ± 0.46 ^bc^	0.47 ± 0.04 ^c^	1.79 ± 0.16 ^ab^	2.22 ± 0.28 ^a^	2.09 ± 0.04 ^a^	1.76 ± 0.03 ^ab^	0.77 ± 0.15 ^c^	0.45 ± 0.22 ^c^	1.08 ± 0.07 ^a^	0.68 ± 0.17 ^c^
*Nramp5*	1.00 ± 0.06 ^abc^	1.15 ± 0.02 ^bcd^	0.26 ± 0.01 ^cde^	0.85 ± 0.06 ^abcd^	0.84 ± 0.05 ^abcd^	1.05 ± 0.22 ^ab^	0.63 ± 0.15 ^de^	0.35 ± 0.01 ^ef^	0.69 ± 0.07 ^cde^	0.79 ± 0.03 ^a^	0.67 ± 0.03 ^f^
*Nramp6*	1.00 ± 0.08 ^cd^	2.8 ± 0.53 ^d^	0.41 ± 0.18 ^cd^	1.30 ± 0.24 ^cd^	3.44 ± 0.55 ^a^	1.91 ± 0.33 ^bc^	0.78 ± 0.12 ^cd^	0.41 ± 0.21 ^d^	0.81 ± 0.16 ^cd^	0.60 ± 0.10 ^ab^	1.50 ± 0.13 ^d^
*HMA2*	1.00 ± 0.02 ^d^	0.75 ± 0.12 ^d^	3.79 ± 0.34 ^a^	2.75 ± 0.50 ^c^	0.88 ± 0.10 ^d^	2.71 ± 0.17 ^c^	0.54 ± 0.04 ^d^	3.88 ± 0.14 ^b^	2.88 ± 0.24 ^c^	0.59 ± 0.01 ^d^	5.41 ± 0.31 ^b^
*HMA3*	1.00 ± 0.03 ^a^	0.72 ± 0.12 ^c^	0.53 ± 0.03 ^c^	0.51 ± 0.07 ^c^	0.43 ± 0.10 ^c^	0.90 ± 0.02 ^a^	0.45 ± 0.08 ^c^	0.59 ± 0.02 ^bc^	0.53 ± 0.02 ^c^	0.53 ± 0.02 ^b^	0.43 ± 0.03 ^c^
*HMA4*	1.00 ± 0.04 ^e^	1.35 ± 0.12 ^d^	4.12 ± 0.26 ^a^	2.45 ± 0.10 ^c^	1.21 ± 0.18 ^de^	1.22 ± 0.01 ^de^	0.94 ± 0.09 ^e^	4.26 ± 0.17 ^b^	2.35 ± 0.01 ^c^	1.56 ± 0.09 ^de^	6.14 ± 0.04 ^b^
*ZIP2*	1.00 ± 0.18 ^d^	39.90 ± 3.34 ^cd^	158.70 ± 34.01 ^a^	36.46 ± 5.61 ^cd^	10.04 ± 1.37 ^d^	29.6 ± 1.57 ^cd^	3.56 ± 0.76 ^d^	87.63 ± 24.82 ^b^	71.05 ± 3.18 ^bc^	1.21 ± 0.51 ^d^	42.38 ± 6.56 ^cd^
*ZIP3*	1.00 ± 0.33 ^ab^	0.88 ± 0.13 ^c^	0.19 ± 0.01 ^c^	0.00 ± 0.00 ^c^	0.26 ± 0.04 ^c^	1.24 ± 0.1 ^ab^	0.43 ± 0.02 ^c^	0.09 ± 0.01 ^c^	1.37 ± 0.04 ^a^	0.17 ± 0.01 ^b^	0.33 ± 0.03 ^c^
*IRT1*	1.00 ± 0.09 ^b^	0.24 ± 0.02 ^cd^	0.09 ± 0.02 ^de^	0.69 ± 0.19 ^bc^	0.29 ± 0.08 ^de^	0.36 ± 0.10 ^cde^	1.73 ± 0.14 ^a^	0.18 ± 0.01 ^de^	0.87 ± 0.16 ^b^	0.49 ± 0.02 ^de^	0.10 ± 0.01 ^e^
*MT2*	1.00 ± 0.02 ^h^	101.51 ± 3.05 ^ef^	212.00 ± 5.11 ^d^	461.15 ± 16.03 ^a^	304.12 ± 59.83 ^b^	308.12 ± 7.02 ^b^	11.97 ± 1.23 ^gh^	274.61 ± 8.26 ^bc^	228.56 ± 13.68 ^cd^	63.76 ± 0.59 ^fg^	149.06 ± 2.98 ^e^

**Table 3:** Moderation analysis of hormone–transporter relationships under H_2_O_2_ inhibition.

Hormone	Transporter	Effect (−Inhibitor)	Effect (+Inhibitor)	Fisher’s r-to-z (Group Diff.)	Interpretation
ABA	*HMA3*	Negative	NS	Significant	H_2_O_2_-dependent suppression of *HMA3*
	*Nramp5*	Negative	Positive	Significant	H_2_O_2_ alters the ABA effect direction
t-Z	*HMA3*	Negative	NS	Significant	H_2_O_2_-dependent suppression of *HMA3*
	*Nramp5*	Negative	NS	Significant	H_2_O_2_-dependent suppression of *Nramp5*
IAA	*HMA3*	NS	NS	NS	No effect on *HMA3*
	*Nramp5*	Positive	Positive	NS	Robust H_2_O_2_-independent stimulation
GA_3_	*HMA3*	NS	NS	NS	No detectable effect
	*Nramp5*	NS	NS	NS	No detectable effect

## Data Availability

The original contributions presented in this study are included in the article/Supplementary Materials. Further inquiries can be directed to the corresponding author.

## Supplementary Materials

The following supporting information can be downloaded at: https://www.mdpi.com/article/10.3390/toxics13100823/s1, Figure S1: Full structural equation model of hormone–ROS–transporter interactions in *S. alfredii*; Table S1: Primer sequences of genes used for this study; Table S2 The concentrations of endogenous hormones in the shoot of *S. alfredii*; Table S3: Leaf cadmium concentrations in *S. alfredii* under different Cd treatments with or without H_2_O_2_ inhibitor; Table S4: Endogenous Hormone concentrations in *S. alfredii* under different Cd treatments with or without H_2_O_2_ inhibitor; Table S5: H_2_O_2_ concentrations in *S. alfredii* under different Cd treatments with or without H_2_O_2_ inhibitor; Table S6: Gene expression in *S. alfredii* under different Cd treatments with or without H_2_O_2_ inhibitor.
