# Supplementary material for: Phytohormone-ROS Crosstalk Regulates Metal Transporter Expression in Sedum alfredii
Source: Toxics. 2025 Sep 26;13(10):823. doi: 10.3390/toxics13100823 (PMC12568203; doi:10.3390/toxics13100823)
Supplement: Supplementary file 1 [file toxics-13-00823-s001.zip › toxics-3872033-supplementary.pdf]

Table S1. Primer sequences of genes used for this study

Genes	Forward primer	Reverse primer
SaACTIN	TGTGCTTTCCCTCTATGCC	CGCTCAGCAGTGGTTGTG
SaNramp1	CGCTGCTGTAGAGGATAAAGAC	CGGGATCAAGATATGCTAGGGA
SaNramp3	AAGAAGCAGCTCATGGGTGT	TAAGCTGCGGTGAAGGTTGA
SaNramp5	GTTGCTCTCATCGGTGGCTCAG	TCGCTGTGGAATTGGCATGTGA
SaNramp6	TGTTTGGCGATTGTGCCAAG	ACATGCCAATTCCACAGCGA
SaZIP1	AGGTGTTATCGGTATCTGCT	ACTTATCCCACGGAGTTTCA
SaZIP2	GATTTGACGGAGAAGGAGTA	GTTTGAAGCGTTGCCTGATG
SaZIP3	TCCCACTCGGTCATTATCGG	TTGGTGTTGTGATGGCGAAA
SaMT2	CTGTGGTTGCGGATCTGCTT	TCCATTCTCCGACACCATCT
SaIRT1	TGCTCCTGCTTCCGTTCA	TGAACGGAAGCAGGAGCA
SaZEP	TGAGCAGGCGGAAGGAATTAGGA	CCTTACCAACACCAATCGCATCCA
SaNCED	CGTGGTACGGCAAGTCGTCTTC	CGCATTCGGATTGGTGGATCAGAA
SaAAO	CGGTCAACTCGTATTCGCTGTCAA	CCACCTTCGCCGCAACTGATT
Sa8OH	CAATATGGTTGCCGATCTCGATGGA	CAAACACAGCCAGGATACCGACTT
SaIAA	TTCGTCGCAGCCGTTACTAGAGA	TTCGCCACAAGAAGTTCAGCAAATG
SaIAAHyd	CCTCCTCCTAGTCACATTCCACCTA	CTTCCTCATCCATTCAACGGTCTCC
SaGA3	GCAGATGATGAATGGTGGTCGGTTA	CCATTGGACAGTGCCATGAAGGT
SatZ	AGCGATGACGACCATACTCTACACT	TGGTGACTCTGACTCCTCGTTGTG
SaSOD	TGACCTTGATGCTCTTGAACCGTTT	TCCACATAATTCCGATGGTGCCTTC
SaCAT	CGGCAGTCAGTGAGTTCTTGTTGT	TGCGGCATCGGTCGTTCATTC
SaPOD	TGTCTCGGTCTCCTCCAGATGC	GCCATTGTTGCGGTGCTGTAGA

Table S2 The concentrations of endogenous hormones in the shoot of *S. alfredii*.

	CK	t-Z		ABA		IAA		GA_3		H_2O_2	
		L	H	L	H	L	H	L	H	L	H
IAA (μg/kg FW)	11.99±0.2a	9.25±0.24b	8.74±0.14b	8.96±0.19b	8.2±0.17b	8.93±0.11b	6.33±0.27d	6.84±0.17d	7.85±0.25c	8.83±0.19bc	11.49±0.44a
ABA (μg/kg FW)	42.86±0.12a	19.16±0.18b	9.14±0.19e	1.04±0.02h	5.59±0.03i	11.01±0.52d	5.64±0.21g	7.59±0.32f	7.93±0.32j	13.28±0.26c	11.52±0.22d
GA_3 (μg/kg FW)	73.98±1.76a	41.51±1de	21.06±0.8gh	40.15±1.43ef	45.52±0.94cd	37.5±1.16f	17.18±0.29hi	15.41±0.69i	24±0.58g	49.83±1.77c	59.03±1.43b
t-Z (μg/kg FW)	42.51±1.06b	29.88±0.78ef	32.83±0.59d	32.76±0.79de	33.11±0.45de	42.02±1bc	40.89±1.02c	41.15±1.66bc	26.04±0.43f	34.65±0.5d	54.3±1.17a

Table S3 Leaf cadmium concentrations in *S. alfredii* under different Cd treatments with or without H_2O_2 inhibitor.

Treatment	Cd Concentration (mg/kg DW)
0.1mM Cd	51.55±0.23f
0.1 mM Cd +5 mM DMTU	112.87±0.59b
0.2 mM Cd	103.78±1.58c
0.2 mM Cd +5 mM DMTU	82.48±0.38d
0.3 mM Cd	59.69±1.39f
0.3 mM Cd +5 mM DMTU	119.43±0.36b
0.4 mM Cd	69.21±0.93e
0.4 mM Cd +5 mM DMTU	82.56±0.63d
0.5 mM Cd	121.02±0.76b
0.5 mM Cd +5 mM DMTU	162.2±0.28a

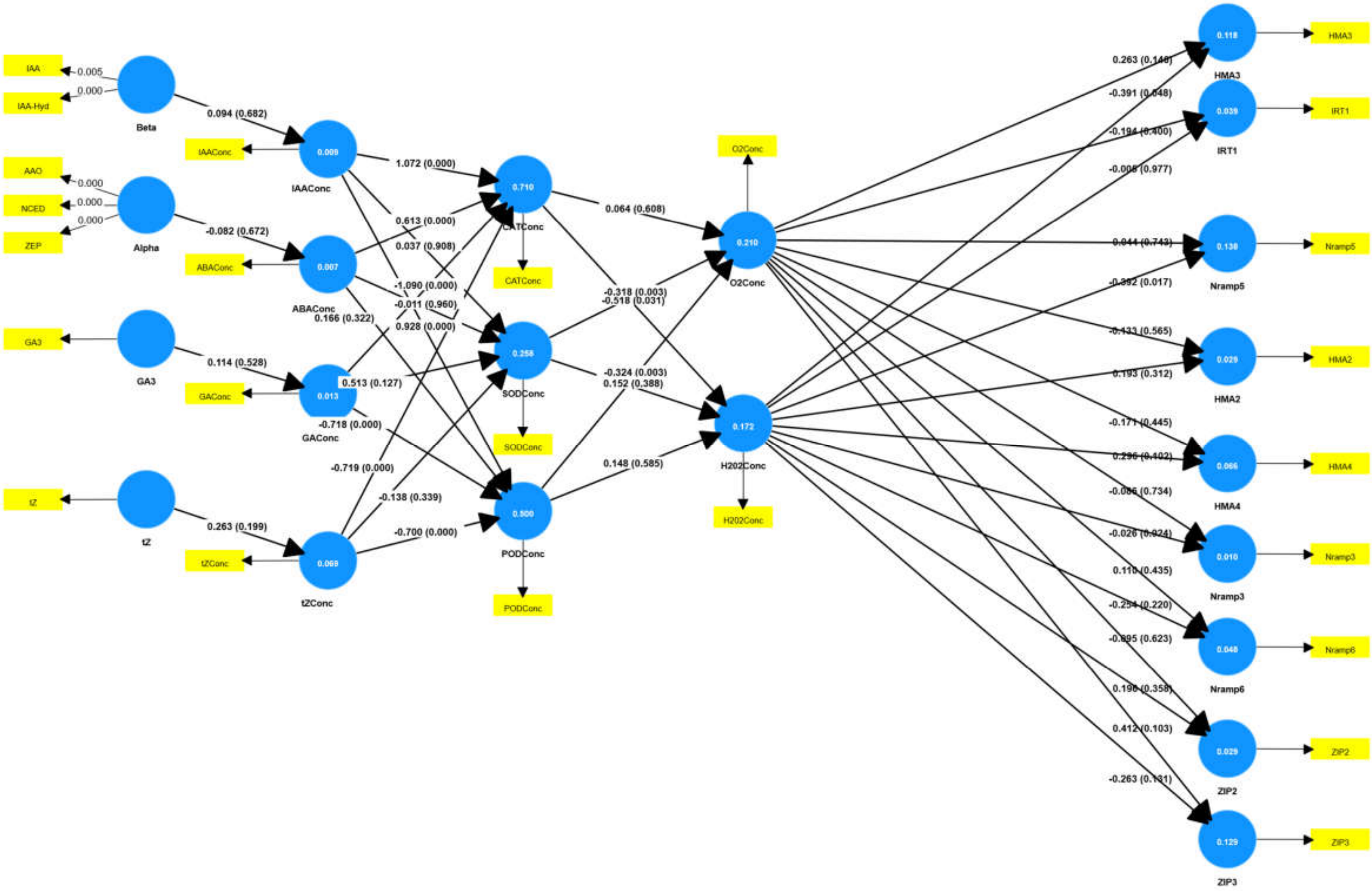

Figure S1 Full structural equation model of hormone–ROS–transporter interactions in *S. alfredii*.

Table S4 Endogenous Hormone concentrations in *S. alfredii* under different Cd treatments with or without H_2O_2 inhibitor.

Treatments	ABA Conc (mg/kg FW)	GA Conc (mg/kg FW)	IAA Conc (mg/kg FW)	tZ Conc (mg/kg FW)
0.1mM Cd	19.65±0.3h	107.64±2.24a	16.08±0.65a	55.91±0.85e
0.1 mM Cd +5 mM DMTU	83.1±1.27c	37.06±1.34de	10.67±0.28c	91.44±3.3cd
0.2 mM Cd	40.32±1.07fg	38.54±1.39d	12.22±0.24b	64.92±1.72e
0.2 mM Cd +5 mM DMTU	44.07±0.67ef	36.25±0.55de	3.96±0.08e	85.54±2.61d
0.3 mM Cd	36.19±0.84g	34.77±0.7de	0.56±0.03f	124.17±2.58b
0.3 mM Cd +5 mM DMTU	93.14±2.99b	38.95±0.39cd	12.75±0.27b	87.48±2.02d
0.4 mM Cd	93.97±2.36b	33.22±0.66e	7.06±0.08d	173.41±8.67a
0.4 mM Cd +5 mM DMTU	100.76±3.08a	26.03±0.3f	12.88±0.52b	93.37±1.43cd
0.5 mM Cd	47.62±0.95de	45.06±2.03b	10.86±0.06c	99.95±3.21c
0.5 mM Cd +5 mM DMTU	51.67±1.37d	42.8±1.73bc	4.39±0.04e	95.34±1.65cd

Table S5 H_2O_2 concentrations in *S. alfredii* under different Cd treatments with or without H_2O_2 inhibitor.

Treatments	H_2O_2 Conc (μmol/g FW)
0.1mM Cd	0.126±0.008a
0.1 mM Cd +5 mM DMTU	0.032±0.002e
0.2 mM Cd	0.064±0.002c
0.2 mM Cd +5 mM DMTU	0.057±0.003c
0.3 mM Cd	0.002±0.001f
0.3 mM Cd +5 mM DMTU	0.085±0.001b
0.4 mM Cd	0.043±0.001d
0.4 mM Cd +5 mM DMTU	0.123±0.001a
0.5 mM Cd	0.006±0.001f
0.5 mM Cd +5 mM DMTU	0.033±0.001e

Table S6 Gene expression in *S. alfredii* under different Cd treatments with or without H_2O_2 inhibitor.

Treatments	*HMA3*	*Nramp5*
0.1mM Cd	1.000±0.015f	1.000±0.006b
0.1 mM Cd +5 mM DMTU	1.278±0.030d	0.003±0.001e
0.2 mM Cd	1.420±0.005c	1.192±0.031a
0.2 mM Cd +5 mM DMTU	1.403±0.022c	0.003±0.001e
0.3 mM Cd	1.286±0.016d	0.003±0.001e
0.3 mM Cd +5 mM DMTU	1.183±0.017e	1.176±0.016a
0.4 mM Cd	2.315±0.048a	0.003±0.001e
0.4 mM Cd +5 mM DMTU	1.142±0.012e	0.795±0.009d
0.5 mM Cd	1.373±0.040c	0.880±0.005c
0.5 mM Cd +5 mM DMTU	1.538±0.010b	0.003±0.001e
